# Genetic Diversity of Human Adenovirus in Children with Acute Gastroenteritis, Albania, 2013–2015

**DOI:** 10.1155/2015/142912

**Published:** 2015-08-03

**Authors:** G. La Rosa, S. Della Libera, S. Petricca, M. Iaconelli, D. Donia, P. Saccucci, F. Cenko, G. Xhelilaj, M. Divizia

**Affiliations:** ^1^Department of Environment and Primary Prevention, Istituto Superiore di Sanità, 00161 Rome, Italy; ^2^Department of Experimental Medicine and Surgery, University of Rome “Tor Vergata”, 00133 Rome, Italy; ^3^Catholic University “Our Lady of Good Counsel”, 1046 Tirana, Albania; ^4^Pediatric Service, University Hospital “Mother Teresa”, 1046 Tirana, Albania

## Abstract

The objectives of the present study were to assess the occurrence of human adenoviruses (HAdVs) in paediatric patients with gastroenteritis in Albania and to characterize HAdV strains. Faecal specimens from children admitted with acute gastroenteritis to the Paediatric Hospital in Tirana were screened for HAdV, using broad-range primers targeting the hexon gene, in combination with species-specific primers targeting the fiber gene. Phylogenetic analysis was then performed to assess the genetic relationships among the different sequences and between the sequences of the samples and those of the prototype strains. Adenovirus DNA was detected in 33/142 samples (23.2%); 14 belonged to species F (13 HAdV-41 and 1 HAdV-40), 13 to species C (1 HAdV-1, 8 HAdV-2, and 4 HAdV-5), 5 to species B (HAdV-3), and 1 to species A (HAdV-12). Rotavirus coinfection was present in 9/33 (27.2%) positive samples. In the remaining 24 positive samples (12 enteric—F species; 12 nonenteric—A, B, or C species), HAdVs were detected as unique viral pathogens, suggesting that HAdV may be an important cause of diarrhoea in children requiring hospitalization. This is the first study investigating the presence of human adenoviruses (species A–G) as etiologic agents of viral gastroenteritis in children in Albania.

## 1. Introduction

Viral gastroenteritis is a serious viral infection, which affects a large number of individuals all around the world. It constitutes a major threat especially to vulnerable individuals such as children, elderly people, and immunocompromised persons [1]. There are several enteric viruses that can cause gastroenteritis. Epidemiological studies have shown that rotaviruses, astroviruses, enteric adenoviruses (serotypes 40 and 41), and caliciviruses family (*Norovirus* and* Sapovirus*) represent the principal causes of acute gastroenteritis occurring in infants and young children. Enteric human adenoviruses cause acute diarrhea sporadically, as well as in outbreaks, and are considered to be the third leading cause of nonbacterial diarrhea among children.

Human adenoviruses (HAdVs) are classified in the family Adenoviridae, genus* Mastadenovirus*, which contains seven known species, from A to G. Fifty-one serotypes have been classified until now by hemagglutination and serum neutralization reaction, but new adenovirus types, including several emerging and recombinant viruses, have been recently identified based on genomic data. To date there are over 60 types of adenoviruses identified, grouped into seven species. HAdVs are double-stranded, linear DNA viruses displaying genome sizes ranging from 34 to more than 37 kb and carrying 40 genes [2]. The disease spectrum includes, besides gastroenteritis, also respiratory, ocular, and urinary tract infections [1]. Although the clinical course is usually mild and self-limiting, infections may cause outbreaks with severe progression, occasionally leading to a lethal outcome even in immunocompetent individuals. Adenovirus has been associated with persistent infections in both immunocompetent and immunocompromised individuals. These infections have the potential to cause fatal disseminated disease. The disease pattern of adenoviruses infection varies according to virus species. Adenovirus species F, types 40 and 41, has been found to be associated with gastroenteritis and is therefore referred to as enteric adenoviruses. Other species such as A, C, and D have also been associated with diarrhoea.

In Albania, gastroenteritis caused by viral etiologies has not been studied thoroughly and no data are currently available regarding the specific contribution of adenovirus in diarrhoeic disease in children.

The specific objectives of this study were to determine the prevalence of adenovirus infections among children with acute gastroenteritis in Albania and to characterize HAdV strains. Accurate understanding of the relative prevalence of the adenovirus in diarrhoea reported cases could be important information for putting into action effective control and preventive interventions.

## 2. Materials and Methods

This study was carried out from October 2013 to January 2015. Faecal samples were collected from children hospitalized at the Pediatric University Hospital “Mother Teresa” in Tirana, Albania, with a clinical diagnosis of acute gastroenteritis. At least three evacuations of liquid or semiliquid stools in a 24 h period were required for making a clinical diagnosis of acute gastroenteritis.

During the study period a total of 142 faecal samples were collected (one sample from each hospitalized child), 72 from female and 70 from male children. The average age of the patients was 14 months, ranging from 2 months to 7 years of age. Informed consent was taken from all children's parents or legal guardians before filling up the relative questionnaire and taking faecal samples for lab examinations.

Viral nucleic acids were extracted from 10% clarified stool suspensions prepared in phosphate-buffered saline, using the semiautomated platform Nuclisens Easymag (bioMerieux), according to the manufacturer's instructions. The extraction process uses magnetic silica-based beads and is based on BOOM technology. Briefly, 400 *μ*L of lysis buffer was added to 200 *μ*L of stool suspension and incubated at room temperature for 10 min. Then 50 *μ*L of magnetic silica particles was added to allow DNA binding. Next, the silica particles were washed twice and wash buffers were removed by vacuum aspiration. Finally, the DNA was recovered from the particles using 100 *μ*L of elution buffer during a 10 min incubation period at 70°C under constant shaking. After the extraction, aliquots of DNA were stored at −80°C and thawed only once.

The collection of faecal samples had been previously investigated for the presence of other enteric viruses (norovirus, sapovirus, enterovirus, rotavirus, and hepatitis A and E viruses) (unpublished data).

Molecular typing assays based on PCR amplification and sequencing of the AdV hexon and fibre genes were used. A portion of the hexon gene was amplified using a nested PCR assay with degenerate primer pair targeting the hypervariable regions HVR1−6 of loop 1, able to amplify all AdV species (universal primers). These primers are able to amplify PCR fragments ranging from 764 to 896 bp in the first PCR, and from 688 to 821 bp in the nested PCR, depending on serotype and species [3]. For amplification of partial fibre gene sequences, published primers based on the conserved regions of this gene were used [4]. The list of primers used in the present study is shown in Table 1. As reference strains for the different HAdV species, we used cultivated clinical strains formerly isolated from hospitalized patients and genetically characterized in a 2006 study [5].

PCR amplification was performed in a 25 *μ*L reaction volume containing 1 *μ*L of each primer (22 pmol *μ*L^−1^) and 2 *μ*L of extracted DNA, using the MyTaq Red Mix Kit (Bioline, UK). A T100 thermal cycler (Bio-Rad) was used with the following settings: denaturation at 94°C for 4 min, followed by 35 cycles of 94°C for 1 min, 45°C for 1 min, and 72°C for 1 min, with a final extension cycle at 72°C for 10 min. After the first round of PCR amplification 1 *μ*L of the volume of the PCR product obtained was used for the second PCR assay using the same conditions. Standard precautions were taken to prevent PCR contamination.

Amplified products were separated on 1* *% agarose gel stained with GelRed. PCR products were purified using a Montage PC* *R*μ*96 Micro-Well Filter Plate (Millipore) and were subjected to automated sequencing (Bio-Fab Research, Rome, Italy).

Bioinformatic analysis was performed as follows: the raw forward and reverse ABI files were aligned and assembled into a single consensus sequence using MEGA software version 6.0. Phylogenetic analysis was performed using the same program.

**Figure 1:**
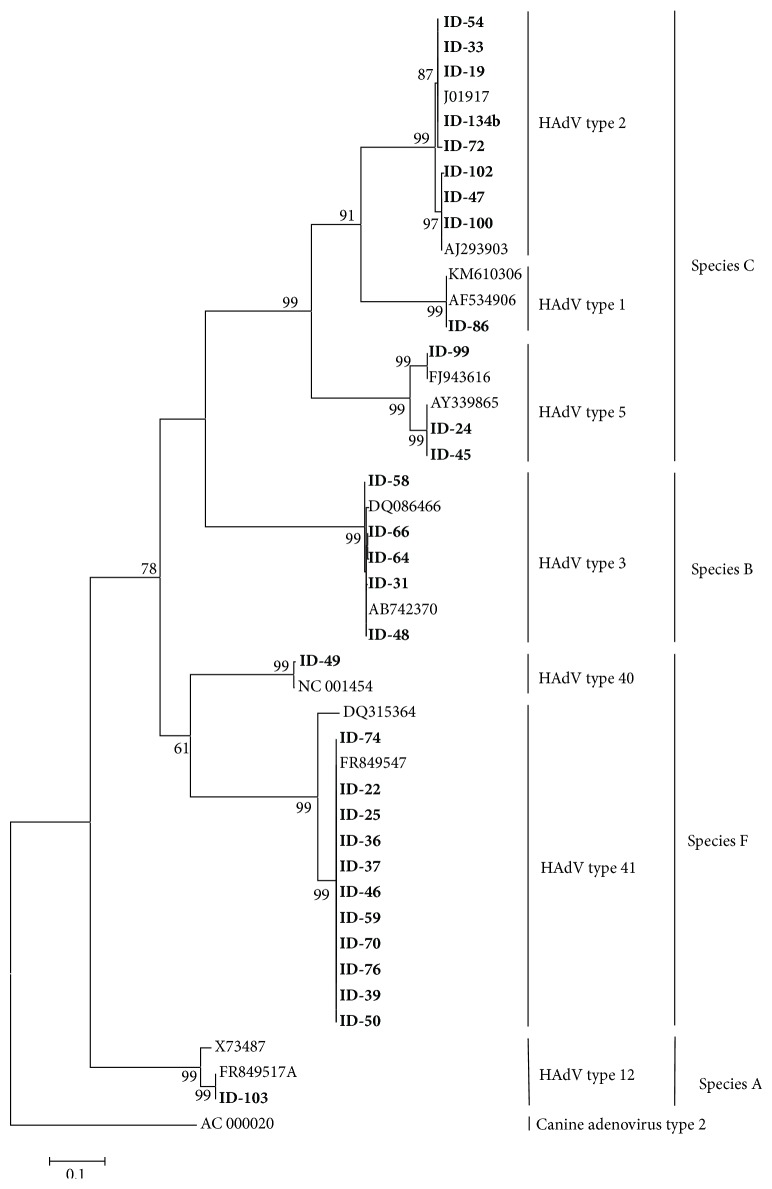
Phylogenetic tree based on partial nucleotide sequences of the hexon region.

The nucleotide sequences were compared to reference adenovirus strains available in the GenBank by BLAST, and molecular type identities were assigned based on the identity of the closest match. The phylogenetic tree was constructed based on the best fit model of nucleotide substitution, which was selected from 24 models available in the software, using the minimum Akaike Information Criterion (AIC). The reliability of the phylogenetic tree was determined by bootstrap resampling of 1,000 replicates. The phylogenetic tree based on nucleotide sequences of adenovirus hexon gene obtained in this study is presented in Figure 1.

## 3. Results

The degenerate primers targeting the hexon gene were able to amplify HAdV DNA in 33/142 samples (23.2%), yielding amplicons of different lengths, depending on the species present in each sample (Table 1). Positive and negative controls yielded the expected results. Seven different types were identified: HAdV-1 (1 sample), HAdV-2 (8 samples), HAdV-3 (5 samples), HAdV-5 (4 samples), HAdV-12 (1 sample), HAdV-40 (1 sample), and HAdV-41 (13 samples). Species F was the most represented, with 14 positive samples (42.4% of the positive samples), followed by species C (13 positive samples, 39.4%). Species B and A were less represented (15.1% and 3% of positive samples, resp.). Coinfections with rotaviruses were detected in 9/33 (27.2%) samples: 6 species C, 1 species B, and 2 species F.

The phylogenetic tree shown in Figure 1 includes the strains sequenced in this work, along with prototype sequences obtained from GenBank. Low-quality sequences from three samples were not included in the tree. Clinical samples (in bold) formed well-supported monophyletic groups and were represented with their corresponding prototype strains within each species. They were grouped into four main clusters, corresponding to species A, B, C, and F, according to BLAST analysis. Species C was further subdivided into three groups, corresponding to types 1 (1 sample), 2 (8 samples), and 5 (3 samples). Species F was subdivided into two groups corresponding to types 40 (1 sample) and 41 (11 samples).

Positive samples detected with the hexon primers were further analyzed for the fiber gene, using the species-specific primers described in Section 2. Twenty-one (out of 33) samples were successfully amplified, producing PCR amplicon lengths consistent with expectations. The fiber gene BLAST analysis was concordant with the hexon analysis for these samples.

The data on age, sex, and major clinical symptoms, along with HAdV typing results, are shown in Table 2.

Median age of children positive for HAdV was 13.5 months (4–28 months). Among the 33 infected patients, 17 were male and 12 female. The information on sex was not available for four children. The two main symptoms of gastroenteritis were diarrhea and vomiting. Of the 14 infants and children found positive for HAdV species F, 5 had diarrhea only (one with bloody diarrhea); the others had both diarrhea and vomiting. The remaining 19 patients found positive for species C, B, and A had both diarrhea and vomiting (15 samples) or diarrhea only (4 samples, one with bloody diarrhea). The average duration of symptoms was 3.7 days (range: 1 to 10) and the average number of evacuations per day was 5.2. The longest 10-day duration was associated with the unique species A sample (HAdV-12).

The seasonal distribution of adenovirus gastroenteritis showed a greater prevalence of infection in April and May (13 cases corresponding to 40% of the total number of positive patients).

## 4. Discussion

Acute gastroenteritis in children, particularly under the age of five, remains a major cause of morbidity and mortality worldwide. In Albania pediatric acute gastroenteritis due to viral etiologies has not been studied thoroughly. The objectives of the present study were to assess the presence of human adenoviruses in pediatric patients with gastroenteritis in hospitalized children and to characterize HAdV strains identified. Samples were tested for HAdV by a PCR assay system targeting the hexon gene. Owing to its hypervariable regions, the hexon region is useful for the classification and recognition of individual serotypes [6]. Indeed, the broad-range primer pair used in this study has been previously found to be capable of amplifying all known HAdVs [4]. Positive samples were subsequently tested using an assay targeting the fiber region. Both the hexon and the fiber genes are considered “hot spots” for base mutations and recombination, which are important driving forces for AdV evolution. Therefore, sequencing the hypervariable regions of the hexon gene and the fiber gene is important for the correct identification and designation of adenovirus strains as demonstrated in previous investigations [4, 7]. In the present study, 63% of the positive samples detected in the hexon region were also positive using the fiber assay, and BLAST analysis was concordant with the hexon analysis for these sequences.

The HAdV-positive rate in children with gastrointestinal symptoms was 23.2% (33/142 samples), which is higher than that found in previous similar studies, showing a prevalence ranging from 2% to 19.6% [8–17]. On the other hand, it should be noted that different and sometimes less sensitive detection methods were used in those studies (including enzyme immunoassay). Moreover, most of the cited papers have addressed exclusively the presence of the “enteric” adenoviruses 40 and 41, while the broad-range assay used in this study is able to detect all known HAdV serotypes. Indeed, a variety of HAdV species (four) and types (seven) were found to circulate among diarrhoeic children in this study. Species F was the most prevalent, followed by species C (mostly HAdV-2). Although species F is frequently found in children with severe gastroenteritis worldwide, nonenteric HAdVs have also been associated with acute gastroenteritis [18, 19]. However, the identified HAdV in the sample should not necessarily be considered to be the causative agent of the infection, as infections caused by members of species C and B can result in prolonged shedding in the faeces after a previous infection [10, 20, 21].

The faecal samples collected for this study had been previously screened for the presence of other enteric viruses. Single HAdV infection was detected in 24/33 cases (72.8%). The only coinfection was found with rotavirus, in 9/33 (27.2% of positive samples). Coinfection adenovirus/rotavirus has been described in other studies on the viral etiology of pediatric gastroenteritis [8, 22, 23]. A dual infection can be explained by the fact that some viruses from a particular episode continue to be excreted while another virus can cause the acute disease. In any case, the determination of the etiological agent that causes the gastroenteritis can be difficult [24]. In this study 7/9 of the coinfections were associated with nonenteric HAdVs, which can probably support the hypothesis that these adenoviruses are not the causative agent of the infection.

However, we cannot rule out the role of species A/B/C adenoviruses in diarrhea etiology, since 12/19 of nonenteric adenovirus types were detected as unique viral pathogens. Indeed previous reports have described an association of adenoviruses with acute gastroenteritis, particularly HAdV-12, HAdV-18, and HAdV-31 (species A), HAdV-3 and HAdV-7 (species B), and HAdV-1, HAdV-2, and HAdV-5 (species C) [10].

HAdV infection occurred throughout the year, peaking in April and May. This pattern is confirmed by some studies about the seasonality of the virus [10], although other studies have reported no seasonal pattern [25, 26].

To the best of our knowledge, only one study on viral gastroenteritis has been published in Albania, addressing the presence of adenoviruses, among other enteric viruses [27]. In that study, published in 2007, enteric adenoviruses were detected in 12.3% of samples, whereas nonenteric adenoviruses were not tested. In the present study, enteric adenoviruses were detected in similar percentages (9.7%); however, nonenteric HAdVs were detected in additional 19 samples (13.4%), 12 of which were unique viral pathogens. These findings highlight the importance of the use of broad-range assays for the detection of all HAdV species, in studies on the viral etiology of gastroenteritis.

In conclusion, the occurrence and molecular characterization of human adenoviruses in pediatric patients with gastroenteritis in Albania are described for the first time in the present study. An accurate understanding of the potential role of HAdV in diarrhea disease is likely to contribute to infection control efforts.

## Figures and Tables

**Table 1:** Primers used in the present study, targeting the hexon and fiber regions.

Primer ID	Sequence (5′-3′)	Amplicon size (bp)	Primer position (5′-3′)^*∗*^	Region	Species
1551	TICTTTGACATICGIGGIGTICTIGA	764–896	19135–19160 (J01917)	Hexon	All
1553	CTGTCIACIGCCTGRTTCCACA		20051–20030 (J01917)		
1554	GGYCCYAGYTTYAARCCCTAYTC	688–821	19165–19187 (J01917)		
1555	GGTTCTGTCICCCAGAGARTCIAG		20016–19993 (J01917)		

1560	TGGAACTTAACAACTGATAT	605	30400–30419 (NC_001460)	Fiber	A
1561	ACTTTCATTGTHATRGGT		31004–30987 (NC_001460)		
1545	ATTCCATCGAGTGCACCTACAC	691	30539–30560 (AY598970)		B
1546	GGGTTGACTCCTGTCCATAAGG		31229–31208 (AY598970)		
1708	TTGTTGCAGATGAAACGCGC	433	31021–31040 (J01917)		C
1709	GTTTGGAGTCTTGCACGGT		31453–31435 (J01917)		
1490	AACTGTGCCTTGGAATCATC	499	29642–29661 (L19443)		F
1491	TTAAGGTTAAAGCCCCGTTT		30140–30121 (L19443)		

^*∗*^The primer positions are based on the GenBank sequence accession numbers indicated in parentheses.

**Table 2:** Summary of HAdV-positive patients' data, including age, date of hospitalization, and main symptoms together with HAdV typing results.

Patient ID	Age/sex	Date of hospital admission	Main symptoms and clinical course	Coinfection	Serotype
19	19 months, female	24/12/2013	Diarrhea and vomit (4 days)	Rotavirus	AdV-2
22	22 months, female	08/01/2014	Diarrhea and vomit (1 day)	No	AdV-41
24	9 months, female	22/01/2014	Diarrhea and vomit (4 days)	Rotavirus	AdV-5
25	6 months, male	22/01/2014	Diarrhea (2 days)	No	AdV-41
31	5 months, male	02/02/2014	Diarrhea and vomit (NA)	No	AdV-3
33	18 months, NA	02/02/2014	Diarrhea and vomit (2-3 days)	No	AdV-2
36	8 months, male	17/02/2014	Diarrhea (6 days)	No	AdV-41
37	18 months, male	17/02/2014	Diarrhea (2 days )	No	AdV-41
39	24 months, male	25/02/2014	Diarrhea (4 days)	No	AdV-41
45	NA	NA	NA	Rotavirus	AdV-5
46	12 months, male	02/04/2014	Diarrhea and vomit (1 day)	No	AdV-41
47	6 months, male	09/04/2014	Diarrhea (4 days)	No	AdV-2
48	18 months, male	16/04/2014	Diarrhea (NA)	Rotavirus	AdV-3
49	8 months, female	17/04/2014	Diarrhea and vomit (7 days)	Rotavirus	AdV-40
50	28 months, male	21/04/2014	Diarrhea and vomit (5 days)	Rotavirus	AdV-41
54B	16 months, male	29/04/2014	Diarrhea and vomit (3 days)	Rotavirus	AdV-2
58	13 months, female	08/05/2014	Diarrhea and vomit (2 days)	No	AdV-3
59	24 months, male	08/05/2014	Diarrhea with blood (4 days)	No	AdV-41
64	18 months, female	15/05/2014	Diarrhea and vomit (2 days)	No	AdV-3
66	4 months, NA	20/05/2014	Diarrhea and vomit (4 days)	No	AdV-3
70	24 months, male	23/05/2014	Diarrhea and vomit (3-4 days)	No	AdV-41
72B	6 months, female	28/05/2014	Diarrhea and vomit (2 days)	Rotavirus	AdV-2
74	11 months, male	29/05/2014	Diarrhea and vomit (7 days)	No	AdV-41
76	11 months, male	05/06/2014	Diarrhea and vomit (3 days)	No	AdV-41
80	24 months, male	06/06/2014	Diarrhea and vomit (3 days)	No	AdV-41
86	9 months, female	04/07/2014	Diarrhea and vomit (6 days)	No	AdV-1
97	8 months, female	06/08/2014	Diarrhea and vomit (2 days)	No	AdV-41
99	NA	NA	NA	No	AdV-5
100	19 months, male	NA	Diarrhea and vomit (3 days)	No	AdV-2
102	6 months, male	25/08/2014	Diarrhea (3 days)	No	AdV-2
103	11 months, female	25/08/2014	Diarrhea and vomit (10 days)	No	AdV-12
112	10 months, female	05/09/2014	Diarrhea with blood (4 days)	No	AdV-5
134B	5 months, female	26/11/2014	Diarrhea (5 days)	Rotavirus	AdV-2

NA = not available.

## References

[1] Lion T. (2014). Adenovirus infections in immunocompetent and immunocompromised patients. *Clinical Microbiology Reviews*.

[2] Robinson C. M., Singh G., Lee J. Y. (2013). Molecular evolution of human adenoviruses. *Scientific Reports*.

[3] Lu X., Erdman D. D. (2006). Molecular typing of human adenoviruses by PCR and sequencing of a partial region of the hexon gene. *Archives of Virology*.

[4] La Rosa G., Iaconelli M., Pourshaban M. (2011). Molecular characterization of adenovirus from clinical samples through analysis of the hexon and fiber genes. *Journal of General Virology*.

[5] La Rosa G., Muscillo M., Iaconelli M. (2006). Molecular characterization of human adenoviruses isolated in Italy. *New Microbiologica*.

[6] Ebner K., Pinsker W., Lion T. (2005). Comparative sequence analysis of the hexon gene in the entire spectrum of human adenovirus serotypes: phylogenetic, taxonomic, and clinical implications. *Journal of Virology*.

[7] Kajon A. E., Dickson L. M., Murtagh P., Viale D., Carballal G., Echavarria M. (2010). Molecular characterization of an adenovirus 3-16 intertypic recombinant isolated in argentina from an infant hospitalized with acute respiratory infection. *Journal of Clinical Microbiology*.

[8] Amaral M. S., Estevam G. K., Penatti M. (2015). The prevalence of norovirus, astrovirus and adenovirus infections among hospitalised children with acute gastroenteritis in Porto Velho, state of Rondônia, western Brazilian Amazon. *Memórias do Instituto Oswaldo Cruz*.

[9] Andreasi M. S. A., Cardoso D. D. D. D. P., Fernandes S. M. (2008). Adenovirus, calicivirus and astrovirus detection in fecal samples of hospitalized children with acute gastroenteritis from Campo Grande, MS, Brazil. *Memórias do Instituto Oswaldo Cruz*.

[10] Pereira Filho E., da Costa Faria N. R., Fialho A. M. (2007). Adenoviruses associated with acute gastroenteritis in hospitalized and community children up to 5 years old in Rio de Janeiro and Salvador, Brazil. *Journal of Medical Microbiology*.

[11] Lekana-Douki S. E., Kombila-Koumavor C., Nkoghe D., Drosten C., Drexler J. F., Leroy E. M. (2015). Molecular epidemiology of enteric viruses and genotyping of rotavirus A, adenovirus and astrovirus among children under 5 years old in Gabon. *International Journal of Infectious Diseases*.

[12] Lu L., Jia R., Zhong H. (2015). Molecular characterization and multiple infections of rotavirus, norovirus, sapovirus, astrovirus and adenovirus in outpatients with sporadic gastroenteritis in Shanghai, China, 2010–2011. *Archives of Virology*.

[13] Mladenova Z., Steyer A., Steyer A. F. (2015). Aetiology of acute paediatric gastroenteritis in Bulgaria during summer months: prevalence of viral infections. *Journal of Medical Microbiology*.

[14] Moyo S. J., Hanevik K., Blomberg B. (2014). Prevalence and molecular characterisation of human adenovirus in diarrhoeic children in Tanzania; a case control study. *BMC Infectious Diseases*.

[15] Osborne C. M., Montano A. C., Robinson C. C., Schultz-Cherry S., Dominguez S. R. (2015). Viral gastroenteritis in children in Colorado 2006–2009. *Journal of Medical Virology*.

[16] Verma H., Chitambar S. D., Varanasi G. (2009). Identification and characterization of enteric adenoviruses in infants and children hospitalized for acute gastroenteritis. *Journal of Medical Virology*.

[17] Bon F., Fascia P., Dauvergne M. (1999). Prevalence of group A rotavirus, human calicivirus, astrovirus, and adenovirus type 40 and 41 infections among children with acute gastroenteritis in Dijon, France. *Journal of Clinical Microbiology*.

[18] Lee J. I., Lee G.-C., Chung J. Y. (2012). Detection and molecular characterization of adenoviruses in Korean children hospitalized with acute gastroenteritis. *Microbiology and Immunology*.

[19] Li L., Phan T. G., Nguyen T. A. (2005). Molecular epidemiology of adenovirus infection among pediatric population with diarrhea in Asia. *Microbiology and Immunology*.

[20] Allard A., Albinsson B., Wadell G. (2001). Rapid typing of human adenoviruses by a general PCR combined with restriction endonuclease analysis. *Journal of Clinical Microbiology*.

[21] Pring-Åkerblom P., Trijssenaar F. E. J., Adrian T., Hoyer H. (1999). Multiplex polymerase chain reaction for subgenus-specific detection of human adenoviruses in clinical samples. *Journal of Medical Virology*.

[22] Raboni S. M., Damasio G. A. C., Ferreira C. E. O. (2014). Acute gastroenteritis and enteric viruses in hospitalised children in southern Brazil: aetiology, seasonality and clinical outcomes. *Memórias do Instituto Oswaldo Cruz*.

[23] Tran A., Talmud D., Lejeune B. (2010). Prevalence of rotavirus, adenovirus, norovirus, and astrovirus infections and coinfections among hospitalized children in Northern France. *Journal of Clinical Microbiology*.

[24] Oh D.-Y., Gaedicke G., Schreier E. (2003). Viral agents of acute gastroenteritis in German children: prevalence and molecular diversity. *Journal of Medical Virology*.

[25] Carraturo A., Catalani V., Tega L. (2008). Microbiological and epidemiological aspects of rotavirus and enteric adenovirus infections in hospitalized children in Italy. *New Microbiologica*.

[26] Wong S., Pabbaraju K., Pang X. L., Lee B. E., Fox J. D. (2008). Detection of a broad range of human adenoviruses in respiratory tract samples using a sensitive multiplex real-time PCR assay. *Journal of Medical Virology*.

[27] Fabiana A., Donia D., Gabrieli R. (2007). Influence of enteric viruses on gastroenteritis in Albania: epidemiological and molecular analysis. *Journal of Medical Virology*.

